# Cortical Correlates of Human Balance Control

**DOI:** 10.1007/s10548-017-0567-x

**Published:** 2017-05-02

**Authors:** Andreas Mierau, Britta Pester, Thorben Hülsdünker, Karin Schiecke, Heiko K. Strüder, Herbert Witte

**Affiliations:** 1 Institute of Movement and Neurosciences, German Sport University Cologne, Am Sportpark Muengersdorf 6, 50933 Cologne, Germany; 2 Bernstein Group for Computational Neuroscience Jena, Institute of Medical Statistics, Computer Sciences and Documentation, Jena University Hospital, Friedrich Schiller University Jena, Bachstraße 18, 07743 Jena, Germany

**Keywords:** Posture, Brain, EEG, Directed functional connectivity, Partial directed coherence

## Abstract

Balance control is a fundamental component of human every day motor activities such as standing or walking, and its impairment is associated with an increased risk of falling. However, in humans the exact neurobiological mechanisms underlying balance control are still unclear. Specifically, although previous studies have identified a number of cortical regions that become significantly activated during real or imagined balancing, the interactions within and between the relevant cortical regions remain to be investigated. The working hypothesis of this study is that cortical networks contribute to an optimization of balance control, and that this contribution can be revealed by partial directed coherence—a time-variant, frequency-selective and directed functional connectivity analysis tool. Electroencephalographic activity was recorded in 37 subjects during single-leg balancing on a stable as well as an unstable surface. Results of this study show that in the transition from balancing on a stable surface to an unstable surface, two topographically delimitable connectivity networks (weighted directed networks) are established; one associated with the alpha and one with the theta frequency band. The theta network sequence can be described as a set of subnetworks (modules) comprising the frontal, central and parietal cortex with individual temporal and spatial developments within and between those modules. In the alpha network, the occipital electrodes O1 and O2 act as a source, and the interactions propagate predominantly in the directions from occipital to parietal and to centro-parietal areas. These important findings indicate that balance control is supported by at least two functional cortical networks.

**Electronic supplementary material:**

The online version of this article (doi:10.1007/s10548-017-0567-x) contains supplementary material, which is available to authorized users.

## Introduction

Balance control is a fundamental component of human every day motor activities such as standing or walking, and its impairment is associated with an increased risk of falling. Controlling posture and balance requires a complex interplay within and between the sensory and the motor systems. Early animal preparation studies suggest upright posture and balance are predominantly controlled by neural circuits in the spinal cord, the brainstem and the cerebellum. However, in humans, there is also strong evidence for a crucial contribution of the cerebral cortex (Jacobs and Horak 2007; Maki and McIlroy 2007; Papegaaij et al. 2014). In two recent experiments, we used electroencephalography (EEG) to identify cortical regions that become activated during a wide range of continuous standing balance tasks differing in difficulty by changing the base of support, surface stability, or both (Hülsdünker et al. 2015), as well as after sudden perturbation of balance (Mierau et al. 2015). The results of these experiments indicate the performance of balancing tasks is associated with significant activation of the fronto-central and centro-parietal cortical regions. Specifically, when balance tasks became more challenging a significant increase in cortical theta activity was observed in the aforementioned regions (Hülsdünker et al. 2015). This is consistent with previous research reporting significant increases in cortical theta activity in response to visually induced postural perturbations (Slobounov et al. 2013), during unstable one leg stance (Slobounov et al. 2009), or walking on a balance beam when compared to normal treadmill walking (Sipp et al. 2013).

The working hypothesis of this study is that cortical networks contribute to an optimization of balance control and that this contribution can be revealed by time-variant, directed functional connectivity analysis between EEG electrodes. Directed functional connectivity, also referred to as effective connectivity (Friston 2003), can be defined as the causal influence of one electrode (or region) on another, where the results are often represented as a network (nodes = electrodes; directed edges = connectivity measures) or a temporally varying network sequence. Time-variant, frequency-selective connectivity analysis is suitable to investigate the temporal dynamics of cortical networks and allows frequency-dependent conclusions about the connectivity structure (Pester et al. 2015). Our study aims to answer the as of yet open question on whether cortical connectivity networks during balancing exist—and if so, how these networks develop in time and which possible functions they have. From previous, above mentioned studies it can be speculated that during balancing on an unstable surface, at a minimum a connectivity network related to theta oscillations with nodes over the fronto-central and centro-parietal cortical regions is involved.

To test this hypothesis, partial directed coherence (PDC), based on time-variant multivariate autoregressive (tvMVAR) models (Milde et al. 2010; Leistritz et al. 2013), is used as a time-variant, directed and frequency-selective interrelation measure (directed interrelation = interaction) that provides several methodological advantages in comparison to other approaches (Winterhalder et al. 2005). Our connectivity analysis is intrinsically linked with a multifaceted investigation of the influential patterns of movement-related artifacts in order to identify time–frequency ranges without or with a low degree of artifacts. This strategy is preferred to straight artifact rejection because the application of all tvMVAR-based connectivity measures to real data leads to the dilemma that on the one hand artifacts can severely affect the results of any analysis, yet on the other hand pre-processing steps such as narrow bandpass filtering and artifact rejection can lead to serious disadvantages in the identification of the network (Leistritz et al. 2013).

According to our working hypothesis, we intend to provide new impetus by testing a novel methodological strategy for the analysis of human balance control. The main information about methods and analysis results are described in the body of the article. In addition, supporting information (SI) is provided which encompasses further details about the analysis strategy, as well as complementary results of our investigations.

## Methods

### Participants, Data Acquisition and Preprocessing

Thirty-nine healthy male university students participated in this experiment. Two subjects were excluded from subsequent data analyses due to technical problems during data recording resulting in excessive artifacts in the EEG signal. Therefore, the data of 37 participants [mean (SD) age: 24.7 (3) years; body weight: 77.3 (8.1) kg; height: 180.4 (5.1) cm; body mass index: 23.8 (2.4) kg/m^2^] were analyzed. All subjects stated that they were: free of injury for at least the last 6 months, had no pain or discomfort and/or were not experiencing any limitation in the range of motion during their daily routine and physical activity. In addition, all participants confirmed that they did not undertake any physical exercise within 48 h prior to the experiment. Participants were informed about the experimental protocol and their written consent was obtained beforehand. The study was designed and performed according to the standards set by the latest revision of the Declaration of Helsinki for medical research involving human subjects, and all procedures were approved by the Ethics Committee of the German Sport University.

The experimental setup is illustrated in Fig. SI 1 A. Each participant completed ten trials of balancing on a stable surface followed by balancing on an unstable surface using the “Posturomed” (Haider Bioswing, Pullenreuth, Germany). The Posturomed (http://www.posturomed.de) is a balance testing and training apparatus equipped with a passively oscillating platform (60 × 60 cm) mounted on eight steel cables. The steel cables are covered by stiff plastic which “progressively dampens/attenuates” the oscillating behavior of the platform in response to external forces (i.e. muscular force). Progressively attenuated oscillating behavior means that the attenuation and thus, the deflection resistance exponentially grows with increasing deflection of the platform. The platform was laterally deflected by 2.2 cm and fixed by an anchor in this position [i.e. balancing on a stable surface (BSS)]. Subjects positioned themselves on their dominant leg in center of the platform. Motor asymmetry was determined beforehand using a standardized inventory (Reiss and Reiss 2000). Each experimental trial lasted a total of 30 s. In each trial the investigator randomly released the anchor within the 5th and 20th second. Mean across subjects onset times did not significantly differ between trials. The anchor release induced a medial movement of the supporting platform and allowed the platform to move freely [i.e. balancing on an unstable surface (BUS)]. The inter-trial interval was set at 1 min to avoid fatigue, and anchor release was synchronized with the ongoing EEG. Onset timing of BUS was determined as the time point following anchor release at which the platform motion exceeded its mean oscillation level in a medial–lateral or anterior–posterior direction during the last 5 s prior to anchor release by five standard deviations. During performance of the task subjects were instructed to place their hands at the iliac crests, focus on a fixation crosspoint at eye level, and to reduce sway to a minimum. EEG was recorded from 32 scalp locations (Brain Products GmbH, Gilching, Germany) overlying the whole scalp and equally distributed over both hemispheres according to the international 10:10-system (Jurcak et al. 2007). One additional electrode was used to measure the electrooculographic signal. The electrical reference and the ground electrode were located on positions FCz and AFz, respectively. The sampling rate was set to 1000 Hz. Electrode impedances were kept below 5 kΩ. The electrode scheme together with corresponding mean recorded time series (averaged by subjects and trials) is depicted in Fig. SI 1B.

The data were first segmented into 8 s epochs based on platform release (i.e. switch from a stable to an unstable surface). Therefore, the last 3 s of BSS and the first 5 s of BUS were analyzed. For connectivity analyses data were re-referenced to the common average reference and ocular artifacts were corrected using the Gratton and Coles ocular correction algorithm (Gratton et al. 1983) implemented in the Brain Vision Analyzer 2 software (Brain Products, Gilching, Germany). Finally, data were downsampled to 100 Hz (Matlab decimate function).

### Time–Frequency Analysis of EEG Activity

The time–frequency analysis of EEG activity aimed at identifying and quantifying oscillatory components of the signal as provided by the resulting amplitude time–frequency maps (TFMs); furthermore the TFMs were used for the detection of possibly occurring artifacts. This time-variant amplitude spectrum analysis was carried out by means of time-variant, multivariate autoregressive (tvMVAR) models and matched Gabor transform (MGT) (Wacker and Witte 2011). MGT is a fusion of the Gabor Transform and the Matching Pursuit approach, which allows amplitude as well as phase extraction with an optimal (individual) time–frequency resolution for each transient signal component. The tvMVAR model estimation was performed by means of the multivariate linear Kalman Filter approach (Milde et al. 2010). In addition to its advantage of time variance, this algorithm separately integrates multiple trials and thus, a loss of information due to averaging by trials prior to or after model estimation is avoided. Consequently, for multi-trial data, it has been demonstrated that this algorithm has an overall superior performance in comparison with other Kalman (Ghumare et al. 2015) and alternative approaches (Milde et al. 2010). The required Kalman control parameters were set to 0.02 (adaption of covariance matrix) and 0.005 (step-width of random walk). The tvMVAR model order *p* was chosen according to Akaike’s information criterion (Akaike 1974) which penalizes both, a poor model fit as well as an excessive number of involved model parameters that have to be estimated. Under further consideration of a sufficient fit between parametric tvMVAR spectra and empirical Fourier spectra, the model order was finally set to *p* = 20. It should be noted, that the same tvMVAR model was subsequently used for the computation of the time-variant PDC.

The tvMVAR model parameters and the MGT both provide TFMs that represent EEG activity (amplitude-TFMs). While the MGT amplitude-TFMs have an optimal time–frequency resolution, the tvMVAR amplitude-TFMs possess a comparable resolution as the subsequently calculated PDC-TFMs and thus allow a more appropriate comparison between time-variant spectrum and time-variant connectivity.

Furthermore, a MGT-based phase locking index (PLI) analysis (Witte et al. 2008) has been used. The PLI analysis yields TFMs indicating the degree of phase-locked oscillations. The ‘sway-related’ interference (i.e. artifact) is observed to varying degrees in all EEG signals, and it is phase-locked to the onset of the BUS condition. Consequently, possibly occurring higher harmonics must also be phase-locked and can be detected via PLI in order to avoid misinterpretations (Wacker et al. 2011). The detailed description of the whole artifact detection and rejection strategy and its results can be found in SI 2.

### Time–Frequency Analysis of EEG Connectivity

The connectivity analysis was conducted by means of time-variant PDC (Baccalá and Sameshima 2001). It is a causal (i.e. directed) connectivity measure that is calculated on the basis of the Fourier transform of the tvMVAR model parameters. In Leistritz et al. 2013, this approach is systematically evaluated with regard to influencing algorithmic parameters (model order, control parameters), effects caused by preprocessing (narrow-band filtering, artifact rejection, number of measurement trials) as well as the modeling approach (bivariate vs. multivariate model), and physiological effects (volume conduction). Such preliminary investigations enable a more reliable PDC analysis and help to avoid misinterpretations of the results.

**Fig. 1:**
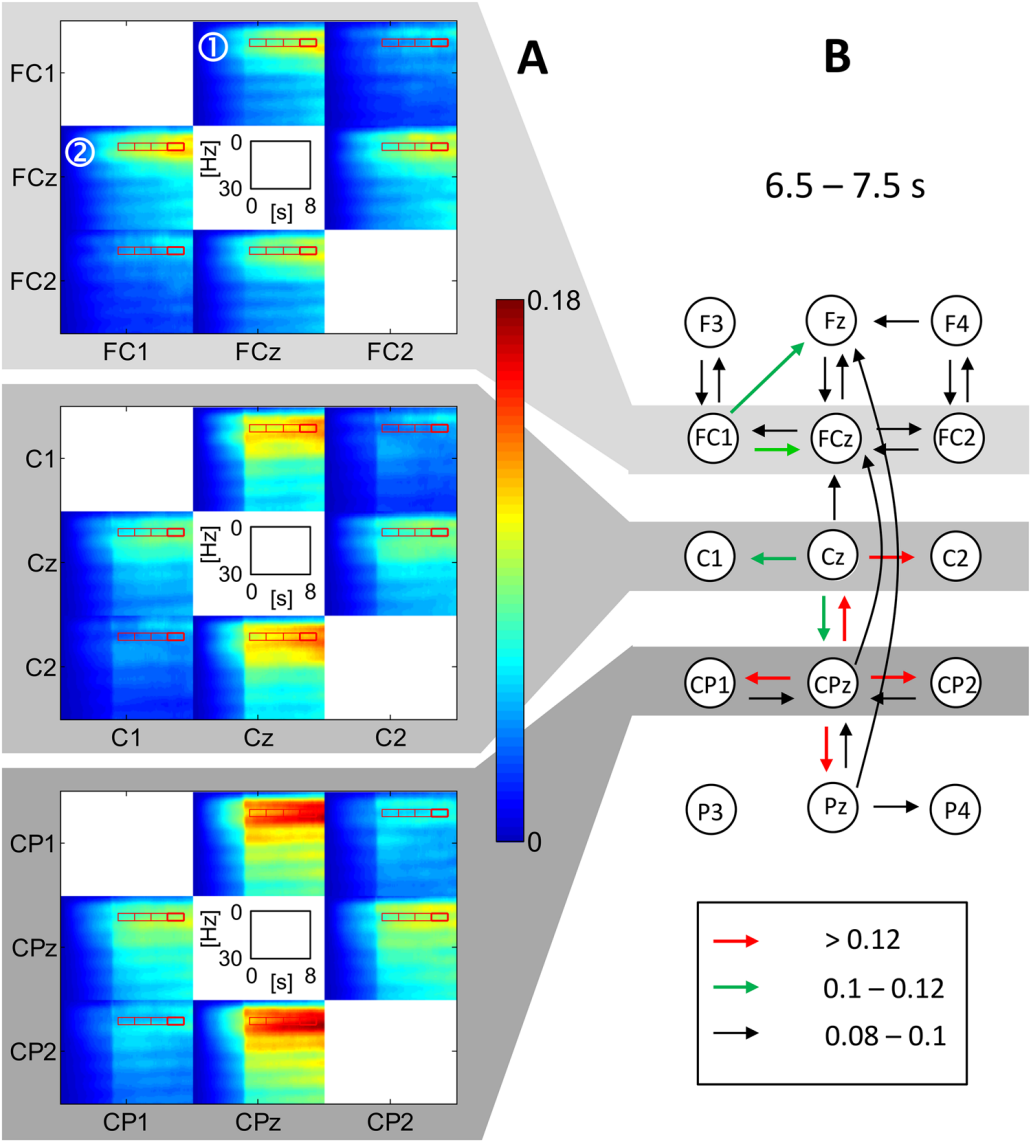
Transition from grand mean PDC-TFMs (**a**) to a weighted directed network of a selected midline electrode array (**b**). The *color* coding represents the PDC value range. To provide a simplified visual analysis, we discretized the weighted networks based on three different mean interaction strengths (0.08–0.1, 0.1–0.12, >0.12). The change from BSS to BUS occurred at time 3 s. (Color figure online)

In order to prevent artificial results due to differing signal amplitudes, time-variant *generalized* PDC (in the following also abbreviated as PDC) was used in this study. It is a scale-invariant development of PDC which is normalized by the model error variances, dispensing any prior signal normalization (Baccalá and Sameshima 2007). For a number of $D$ electrodes the results of PDC computation can be represented as a $D \times D$ block-matrix (PDC matrix) with zero diagonal elements. This consists of $D \cdot (D - 1)$ PDC-TFMs $(x{\text{-axis}}={\text{time}}, y{\text{-axis}}={\text{frequency}}, z{\text{-axis}}\,({\text{color}})={\text{PDC } \text{values}} \overset{\wedge}{=}{\text{interaction } \text{values}}).$ In Fig. 1a several sub-matrices of the grand mean (i.e. mean over subjects) PDC matrix are depicted. Each PDC-TFM comprises all information on the interactions for one of two directions between a pair of electrodes (e.g. FCz → FC1, designation ①, Fig. 1a) and the corresponding PDC-TFM of the reverse direction (FC1 → FCz) is represented by the symmetric matrix element (designation ②, Fig. 1a). The PDC results can be transferred into a weighted, directed network that is composed of all electrodes and the interaction values (PDC) between them (Fig. 1b). As a scalar interaction measure, the mean within a region of the PDC-TFMs is used (e.g. red solid frame), where a region is defined by a frequency band $\Delta f$ and a time interval TI of interest (as an example Fig. 1b: $\Delta f=5{-}7\,{\text{Hz}},$ TI = 6.5–7.5 s). Both, $\Delta f$ and TI values constitute the regions of interest (ROI) from which the mean PDC is extracted. For better visual analyzability, disjunct value ranges of mean PDC values (0.08–0.1, 0.1–0.12 and >0.12) are used for network construction. During BSS the highest mean PDC for the theta network is around 0.08 whereas during BUS it is around 0.18. Based on this information, the PDC’s discretization ranges have been defined. In this way the time evolution of the network weights (mean PDC within an ROI) can be studied as an overlay of weight-related subnetworks (red, green, and black arrows in Fig. 1) for each analysis interval.

The PDC connectivity analysis can be condensed to essential characteristics by calculating basic network measures (Rubinov and Sporns 2010). One appropriate measure for quantifying the global degree of connectivity is the average node strength (ANS) (Barrat et al. 2004) which can be used to demonstrate significant connectivity differences between BSS and BUS for each frequency bin (see “Statistics” section). The node strength of one node quantifies the general level of communication between this node and the other nodes of the system and is defined as the sum of all incoming and outgoing weights associated to this node. The ANS of the network is then gained by the arithmetic mean of all node strengths and provides a measure of the global connectedness within the whole system. The results of ANS analysis are based on the computed PDC-TMFs without discretization.

### Tensor Decomposition

Because of the massive production of data resulting from PDC analysis, tensor decomposition was proposed (Pester et al. 2015). This application decomposes the multivariate PDC output into a sum of rank-1 outer products (Kolda and Bader 2009) which leads to a significant reduction in the volume of results, thereby considerably simplifying the identification of general connectivity patterns and eliminating the requirement for any preliminary definition of spatial/temporal/frequency ROIs. Furthermore, it enables a straightforward integration of multiple subjects without the need for a subject-by-subject analysis or a prior averaging over the group.

### Statistics

**Fig. 2:**
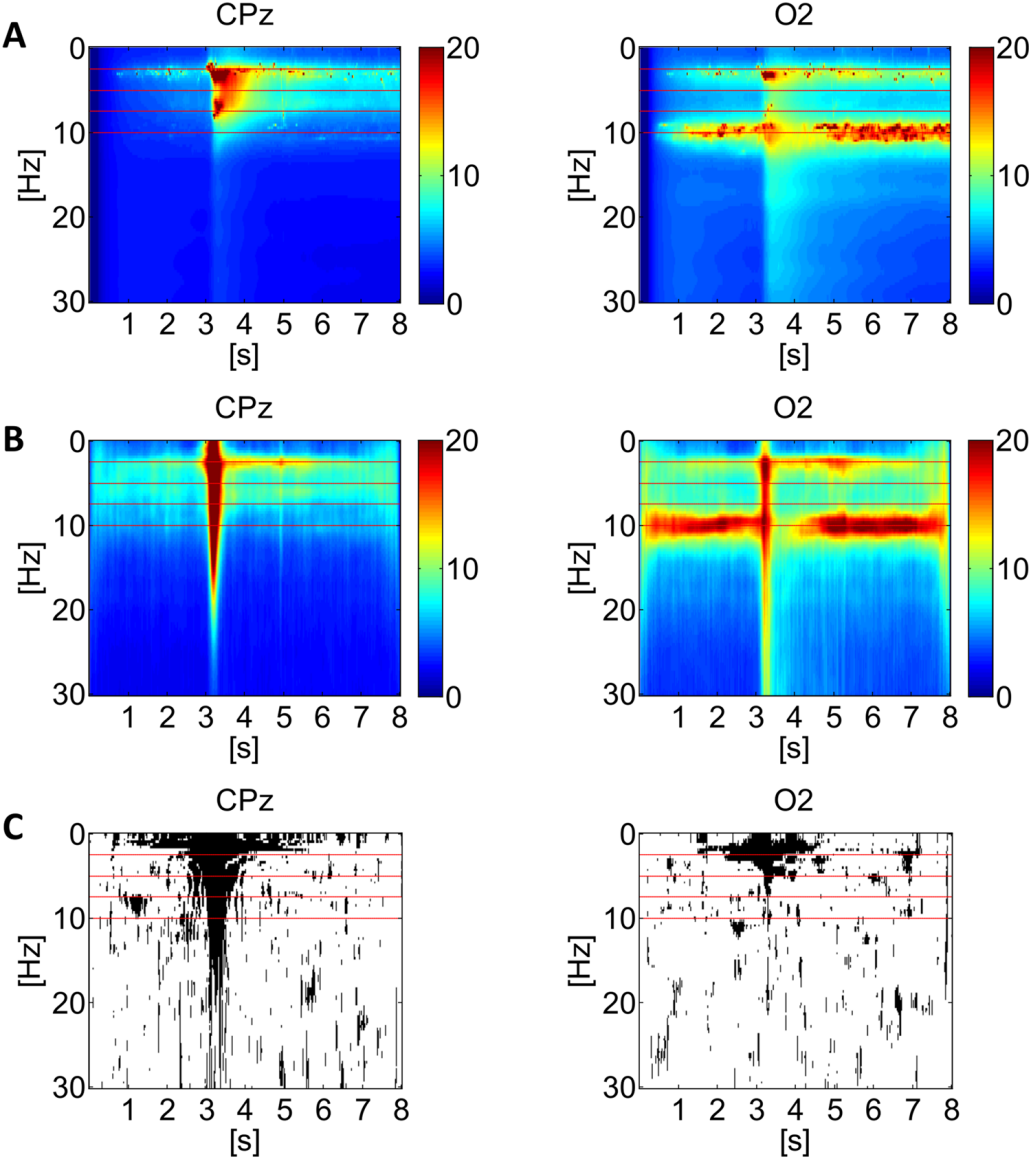
Selected results of the grand mean time–frequency analysis (amplitude spectrum) for the EEG at CPz and O2. The TFMs in *row* (**a**) are based on our tvMVAR approach and those in *row* (**b**) on MGT. The *color* coding represents absolute amplitude values (µV). The TFMs of significant PLI-values (significance level = 0.05) are depicted in *row* (**c**) and designated by *black color*. The *horizontal red dashed lines* designate 2.6, 5.2, 7.8, and 10.4 Hz (possible harmonics of the sway-related interference). The change from BSS to BUS occurred at time 3 s. (Color figure online)

The non-corrected Rayleigh test was used to create a trigger threshold for the detection of time segments of strong phase locking in a PLI time–frequency map (Fig. 2c). The null hypothesis is that the phases are uniformly distributed. Under this hypothesis, the PLI tends to zero. The application of the statistical threshold results in binary PLI-maps (0 = below the threshold or 1 = above the threshold). In this study, the significance level for thresholding was *α* = 0.05.

In order to test whether there is a significant difference of frequency band activity between BSS and BUS situation, a Bootstrap approach was used. For each channel and the theta and alpha frequency band, the confidence tube was estimated based on 1000 Bootstrap replications, where the 2.5% quantile defined the lower bound, and the 97.5% quantile defined the upper bound of the confidence tube. A detailed description of the method is provided in SI 2.

A statistical difference between global connectivity during BSS vs. BUS was confirmed as follows: for each frequency bin, the PDC networks were averaged across time during the BSS and the BUS period separately; and for the resulting two networks, ANS was calculated as a measure of global connectedness. In order to test whether there is a significant change of frequency-dependent connectivity between BSS and BUS, a paired *t* test across subjects has been used. Common type I error level was set to α = 0.05 and was adjusted to multiple testing by Bonferroni correction.

## Results

### Time–Frequency Analysis of EEG Activity

The grand mean amplitude-TFMs of activity showed strong theta oscillations at about 6 Hz (e.g. CPz) and alpha oscillations at 10 Hz (amplitude-TFMs based on the tvMVAR model and matched Gabor transformation). The results of the confidence tube analyses can be summarized as follows: in particular at CPz the mean amplitude within the theta sub-band was increased about 2 s after the onset (from about 7.5 to 10 µV) to the BUS situation. For the electrodes CPz and Pz (5–8 s) as well as Cz, C1, CP1, CP2 (5–6 s) the mean theta amplitude significantly increased during BUS compared to BSS. For each of these electrodes no overlap of the confidence tubes (95% confidence interval) for BSS and BUS could be observed, meaning that there is a significant difference in activity between these two balancing conditions in the theta band. In addition, strong alpha amplitude suppression (‘alpha drop’) occurred at the occipital electrodes and, in a weaker form, at the parietal electrodes (Fig. 2b). This ‘alpha drop’ reaches its maximum about 1 s after the onset of BUS. A significant difference of the alpha amplitude between BSS and BUS, after the ‘alpha drop’, was not observed. Examples of the confidence tube analysis are illustrated in Fig. SI 2.2.

The results of this detailed time–frequency analysis were also used to investigate whether and, if so, in which frequency bands (ROI) a connectivity analysis is appropriate. The results are described in detail in SI 2 and can be summarized as follows: (1) for the theta and the alpha band a contamination by higher harmonics of the ‘sway-related’ interference is unverifiable, so that a connectivity analysis within the chosen frequency bands is meaningful; (2) the time-interval ranging from 3 to 3.5 s is strongly influenced by the BUS onset (motion artifact). This is also true for the theta and alpha band. According to the time-resolution of the tvMVAR-based analysis, the artifact influence is detectable until 1 s after the BUS onset, i.e. the interpretation of this fact must be considered.

### Connectivity and Network Analysis

According to the results of activity-based time–frequency analyses, the regions of interest with regard to electrodes, temporal samples, and frequencies for our PDC-based network analysis were chosen. The mid frequencies of the selected frequency bands, which include a theta (5–7 Hz) and an alpha (9–11 Hz) sub-band, correspond to the grand mean results of the time–frequency analysis. For the network representation of our results, the following ROIs 1–4 were defined according to time intervals: TI1 = 3.5–4.5 s, TI2 = 4.5–5.5 s, TI3 = 5.5–6.5 s, TI4 = 6.5–7.5 s. The procedure illustrated in Fig. 1 was applied for each sub-band to each TI which leads to two sequences of four weighted, directed networks that represent the connectivity properties during the BUS for both the theta and alpha networks (sequence).

In a preliminary step the influence of remaining artifacts on the connectivity analysis was investigated. The conclusions drawn from these investigations are that interactions which are originated from the electrode sites Fp1 and Fp2 should be excluded from an interpretation and those related to the sites T7, T8, P7, and P8 must be interpreted carefully. Furthermore, the influence of artifactual EEG activity on the central, centro-parietal, parietal and occipital network interactions is only marginal. According to our working hypothesis that the connectivity networks are presumably located in these brain areas, the full tvMVAR model has been used for further investigations.

**Fig. 3:**
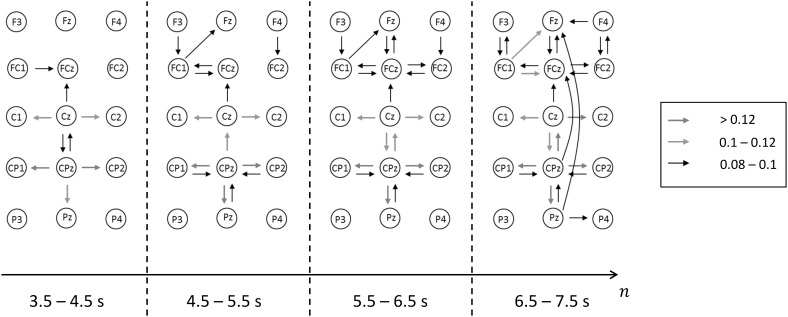
Dynamics of the theta network (5–7 Hz) after* onset* of BUS. The *color* coding of the PDC-TFMs are discretized in three ranges (*red, green* and *red arrow*). (Color figure online)

The results for the connectivity analysis for the BSS and BUS are reported here, but those for the BSS are not illustrated. For network analysis the lowest discretization level for the mean PDC values was chosen in such a way that for the theta networks during BSS no strong interactions exist. For better illustration of the results, only the PDC ranges 0.08–0.1, 0.1–0.12 and >0.12 are used (Fig. 3), representing the strongest interaction degrees. The theta networks formed by the strongest interactions are depicted for all electrodes in Fig. SI 2. These results provide a clear rationale for using a limited set of electrodes for presenting the networks in Fig. 3, i.e. the theta network topography for the strongest interactions is restricted to these electrodes. This is also true for the alpha network topography, which is restricted to another but also topographically circumscribed electrode set.

For the description of the network structure we use the term module, which is defined as a set of nodes that maintain a large number of interactions amongst each other (intra-module edges) and a small number of interactions to nodes outside their module (inter-module edges) (van den Heuvel and Sporns 2013). For the BUS-related theta band network sequence it can be clearly demonstrated that CPz and the surrounding electrodes constitute a module. CPz acts as a central source electrode, where the interaction effect is topographically limited. The electrode site Cz is also involved and forms a second theta subnetwork together with CPz. A further module is formed by the electrode sites FCz, FC1, and Fz. The interactions in the frontal, fronto-central sub-module are not as strong as in the sub-module CPz/Cz and those between both sub-modules are even lower. The basic structure of the theta network can already be observed right after the onset of BUS (time interval 3.5–4.5 s). However, the interactions become stronger with increasing temporal distance to the onset. The module structures and their changes can also be obtained by using the extended theta band (4–8 Hz) and different bands e.g. 6–8 Hz (not documented in this article). As already mentioned above, another important finding is that during BSS (baseline) no interactions with (mean) PDC values >0.08 can be observed, i.e. these module structures are unique to BUS.

**Fig. 4:**
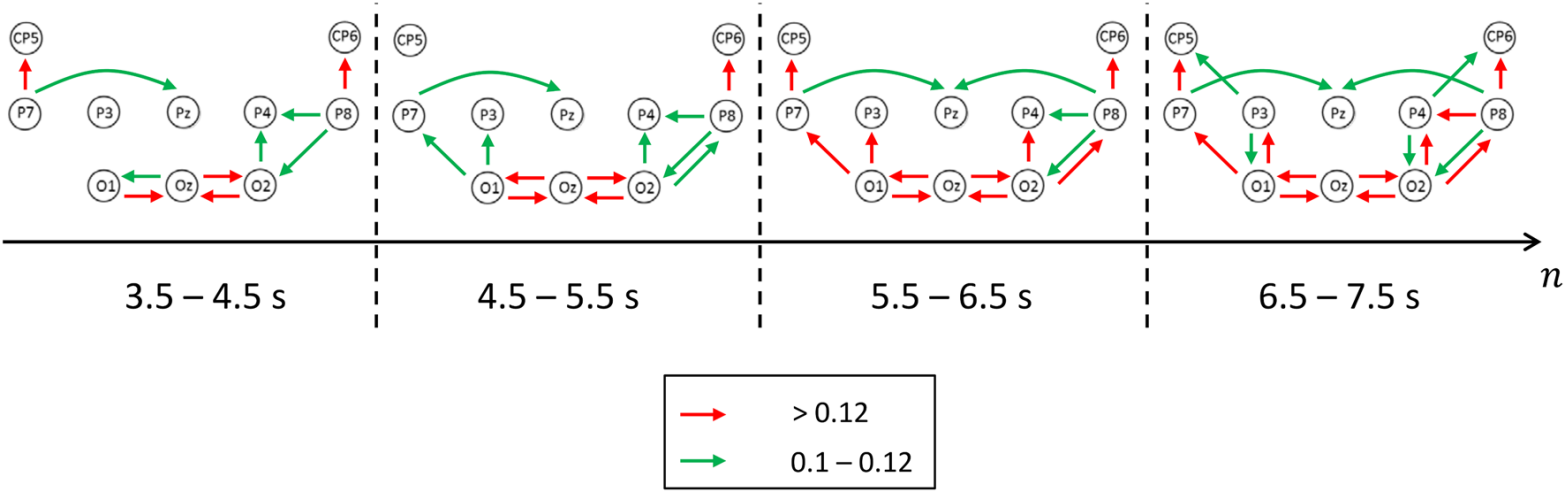
Dynamics of the alpha network (9–11 Hz) after* onset* of BUS. The *color* coding of the PDC-TFMs are discretized in two ranges (*red* and *green arrow*). (Color figure online)

In the same way, the sequence of alpha networks was investigated. Figure 4 illustrates the results for the TI1–4, where only two discretization steps were used (0.1–0.12 and >0.12). For BSS (not shown), the strongest interactions can be observed between the occipital electrodes with O1 and O2 acting as sources for Oz. For TI4 during BUS this network structure shows an expansion towards the left and right parietal as well as centro-parietal electrode sites (P7/8, P3/4, CP5/6). This expansion encompasses large parts of the magnocellular and parvocellular systems, which are hypothesized to form two subsystems within the pathway for processing of visual information (Vialatte et al. 2010). Specifically, the interactions between CP5/6, P7/8, O1/2/z are suggested to reflect the parvocellular system and those between P3/4, O1/2/z the magnocellular system. The most noteworthy point is that the nodes O1 and O2 act as a source, and that the interactions propagate predominantly in the directions from occipital to parietal and to centro-parietal areas. The midline areas (i.e. Pz/CPz) are clearly omitted. A topographical overlap with the theta network does not exist for the two strongest interaction degrees (i.e. red and green arrows in Figs. 3, 4). The network based on TI1 is very similar in comparison to the later networks. This means that the ‘alpha drop’ (see above) is not associated with a specific network structure.

Finally, we found a significant global increase of the degree of connectivity within the PDC networks during BUS as compared to BSS: the paired *t* tests for ANS values during BSS as compared to during BUS yielded *p* values below the significance level of *α* = 0.05 (Bonferroni corrected with respect to considered frequencies) across the entire frequency range 0–30 Hz. That means, compared to the BSS task, the BUS task leads to an enhanced connectedness within the whole network, irrespective of the considered frequency.

### Tensor Decomposition

The set of subject-related PDC matrices forms a fourth order tensor (PDC tensor) with the modes space, time, frequency, and subject, i.e. a 4-mode tensor decomposition enables a subject-wise investigation with a reduced dimensionality of results without using regions of interests with respect to topography, time or frequency. The application of a 4-mode parallel factor analysis (PARAFAC) with *M* factors results in *M* spatial matrices (mode space) that indicate the strength of interactions concerning all electrode combinations, in *M* time courses (mode time) that indicate the time-variant changes of connectivity, in *M* frequency loadings (mode frequency) that indicate the frequency-dependent variability of network patterns, and finally in *M* subject-related loadings (mode subject) that indicate the individual differences between the subjects. The handling and interpretation of these factors is then considerably easier and more intuitive than the evaluation of raw, subject-related PDC matrices.

We performed both, full-node (PDC matrix with all PDC-TFMs, not shown here) PARAFAC analysis, as well as the decomposition of a tensor with a reduced number of electrodes, namely electrodes which have turned out to be particularly involved in the theta and alpha network. It is important to note that the full tvMVAR, applied first, is used to derive the PDC-TFMs under consideration of the whole set of electrodes. Then, the tensor decomposition of the subset PDC-TFMs is aimed to reveal subset-immanent connectivity patterns.

**Fig. 5:**
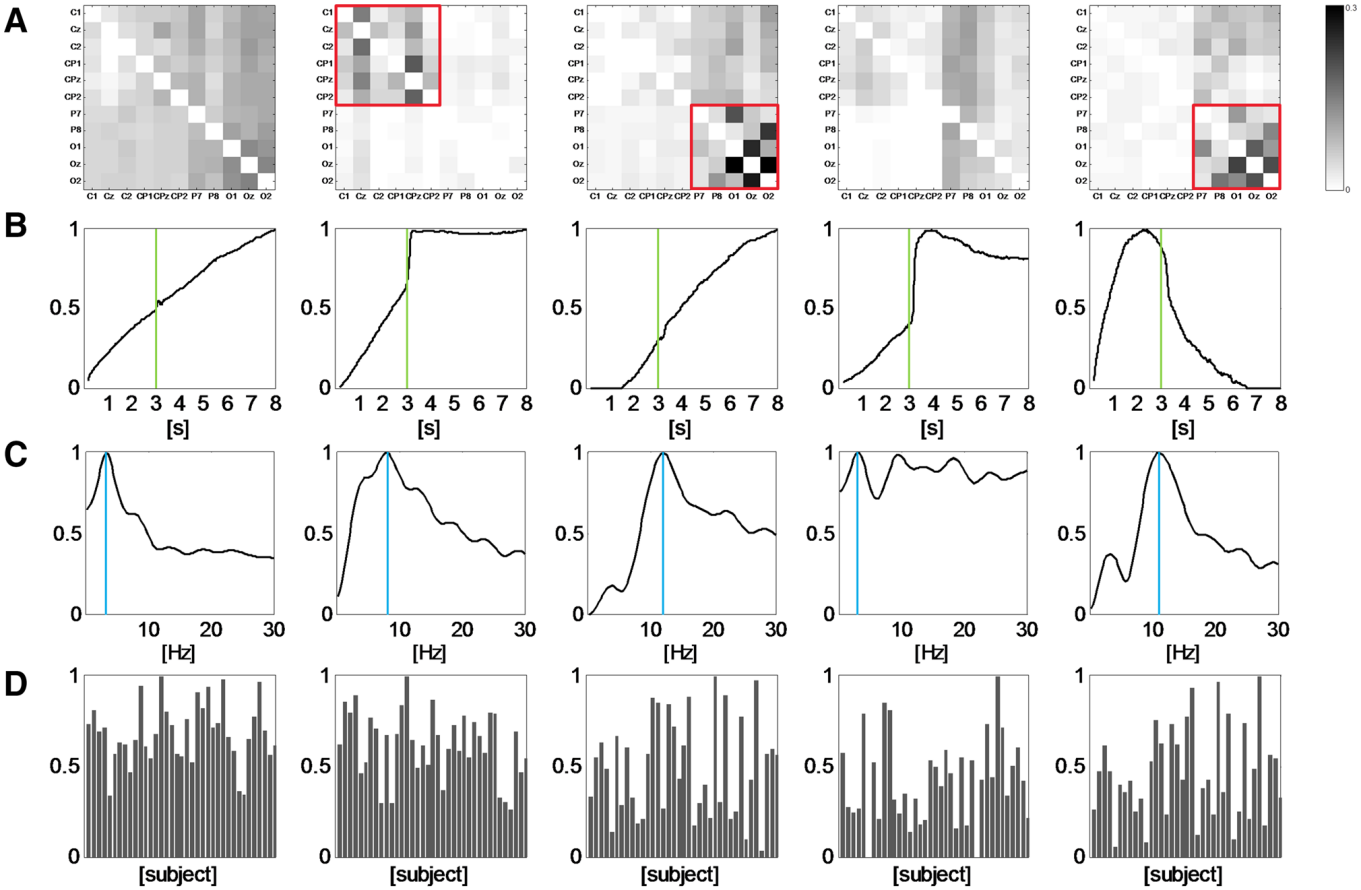
Results of the tensor decomposition. **a** The spatial maps (where are the connections of this factor?) comprising the channels C1, Cz, C2, CP1, CPz, CP2, P7, P8, O1, Oz, O2. The *red* frames designate the electrodes which clearly form separate modules within a common factor. **b** The temporal development (when do the connections of this factor occur?). The *green lines* at time point 3 s indicate the transition from BSS to BUS. **c** The spectra (at which frequencies do the connections of this factor occur?). The *blue lines* point out the frequency where the loadings reach the maximum for a given factor. Finally, **d** shows the subject-wise loadings (which subjects share this factor?). Every *column* represents one factor, i.e. spatial map, time course, spectrum and frequency loadings of one column correspond to the same factor. (Color figure online)

Figure 5 shows the results of a 4-mode tensor decomposition with $M=5$ factors for a partial network, which comprises the main nodes of the theta and alpha networks (C1, Cz, C2, CP1, CPz, CP2, P7, P8, O1, Oz, O2). Factor #1 (1st column) can be clearly attributed to the sway-related interference at 2.6 Hz (mode C) in all selected electrodes (mode A) and subjects (mode D). The proportion of this specific mode to the network strength increases over time (mode B). Factor #2 (2nd column) describes the circumscribed theta network (red frame in the 1st row, 2nd column) which occurs after the onset of BUS (green line in the 2nd row, 2nd column) and remains thereafter. The frequency mode (3rd row, 2nd column) has a peak maximum at the border between the theta and alpha bands. This is true for the vast majority of subjects (mode D). Factor #3 represents the alpha network, which is clearly visible in the topography mode (red frame in the 1st row, 3rd column) as well as in the corresponding frequency mode (blue line in the 3rd row, 3rd column). The time mode (Fig. 4b, 3rd column) indicates that the presence of the alpha network related to this factor increases during both, BSS and BUS. The subject mode shows more variability as compared to the first two factors. The strong, impulse-like artifact at the onset of BUS (release of the platform anchor) is reflected by factor #4 (2nd row, 4th column), where all frequencies are involved (3rd row, 4th column). Factor #5 represents the topography of the alpha network (red frame in the 1st row, 5th column) during BSS (2nd row, 5th column). The corresponding peak in the frequency mode of this alpha network is about 11 Hz and thus, slightly lower than the frequency peak around 12 Hz of factor #2 (3rd row, 2nd column vs. 3rd row, 5th column). It must be noted at this point that the interpretations of a frequency mode and of a spectrum are different because the frequency mode is a result of a mathematical decomposition procedure for the connectivity analysis results (PDC-TFMs), rather than a representation of spectral properties of the recorded EEG data.

## Discussion

### Theta Network

The theta network occurring after the transition from BSS to BUS is associated with a notable increase in activity (static balancing as baseline) at a few network electrodes in proximity to the CPz. This network comprises more electrodes than those with a significant theta activity increase. However, theta oscillations derived from CPz (see Fig. 2) and Pz (not shown) are characterized by a circumscribed trace within the amplitude-TFM. It can be assumed that activity recorded by these electrodes contain activity from the midline of the posterior somatosensory association cortex (SAC). The SAC is found within the parietal cortex and it is directly interconnected with the (pre)motor cortex, including the frontal eye field which is crucially involved in the control of eye movements (Kandel 2013). Among other important higher order central functions, the SAC is engaged in somatosensory integration (Iwamura 2003) and visuomotor coordination (Georgopoulos and Grillner 1989). This is consistent with our hypothesis that a theta network may contribute to an optimization of the balancing control and our results concerning theta activity confirm our previous investigation (Hülsdünker et al. 2015), which show a significant increase of theta activity in fronto-central and centro-parietal cortical areas with increasing balance task demands. All our results confirm the results from Sipp et al. (Sipp et al. 2013), who investigated the localization of the theta band response related to the loss of balance during balance beam walking. These authors reported that if subjects lost their balance and stepped off the narrow treadmill-mounted balance beam, then a significant increase of theta activity within the cortical areas occurred that have projections to electrodes constituting the nodes of the theta connectivity network identified by this study.

The centrally located theta network module around CPz is already established around one second after the onset of BUS and remains stable until the end of the analyzed period (5 s). It is striking that this module remains mostly ‘undisturbed’ from interactions of other electrodes, i.e. it is almost isolated. Additionally, a strong directed interaction towards Cz develops during BUS (about 1.5–2 s after the onset), which itself acts as source for neighboring nodes (C1/2). This could mean that the involvement of the primary motor cortex within this network occurs after involvement of the SAC, which highlights the advantages of the time-variant approach in contrast to stationary methodologies. The somatotopic representation of the feet and legs comprises the medial part of the primary motor cortex M1 and that of the neck and trunk comprises the more lateral parts. Coinciding with the beginning of interactions related to M1 a frontal module (nodes FCz, FC1/2, Fz, F3/4) evolves over the supplementary motor area (SMA) and the premotor area (PMA). Associated functions are e.g. motor sequencing and planning as well as movement initiation and inhibition (Nachev et al. 2008). Towards the end of TI4, weak interactions from parietal nodes to nodes of the frontal module (Pz → FCz, CPz → Fz) can be observed. This suggests the relevant sensory information sent to the medial frontal cortex becomes an integral part of balance control once the individual experiences the initial balance disturbance, and consequently more finely tuned motor responses are needed to meet demands of the task (i.e. keep platform movements to a minimum). Interestingly, no interactions were found from SMA/PMA to M1. The results of tensor decomposition clearly show that BUS is associated with a development of centro-parietal theta network in each of the 37 subjects (factor #2).

### Alpha Network

According to the ‘gating function’ theory for alpha activity, higher alpha amplitudes can be predicted in inhibited cortical areas and lower amplitudes (e.g. during ‘alpha drop’) in the cortical areas involved in information processing (Toscani et al. 2010), i.e. ‘alpha drop’ can be interpreted as loss of inhibition. This effect is most pronounced at the occipital (O1/2/z), parietal (P3/4/7/8/z) and temporal electrodes (T7/8) and may reduce the inhibitory function of alpha activity during the first second after onset of BUS. It can be speculated that the transient ‘alpha drop’ acts as an ‘initial boost’ to establish the theta and/or the alpha network; in general, such interplay between release from and recovery of inhibition can be considered as an active process for supporting information processing. The ‘alpha drop’ effect can also be observed at the beginning of other postural or balance control situations (Slobounov et al. 2008).

The electrodes of the alpha connectivity network cover those of the centro-parietal theta network like a hull, and almost no topographical overlaps (see below, specialty of Pz) between both networks exist. In contrast, strong oscillatory alpha activity can be detected at electrodes over the whole parietal lobe. During BSS a narrow circumscribed network already exists (O1 → Oz, O2 → Oz, not shown). However, it spreads out topographically after the onset of BUS, and its interactions become stronger over time. This spreading includes exactly those temporo-parietal (also centro-parietal) electrodes at which the ‘alpha drop’ (amplitude suppression) is observed (except for Pz). In its final state (TI4) this network encompasses large parts of the assumed ventral and dorsal pathways of visual information processing (Vialatte et al. 2010), where the main direction of interactions (processing) is clearly from the occipital to the temporo-parietal cortical areas (O1 → P3, O2 → P4; O1 → P7 → CP5, O2 → P8 → CP6). This finding indicates that both the ventral (also known as focal or ‘what’ system—object motion perception and object recognition) and the dorsal (also known as ambient or ‘where’ system—e.g. sensitive to movement scene) visual system are probably involved into the optimization of balancing control. Vision is not necessary for normal balance. However, postural stability is improved by vision (Guerraz and Bronstein 2008). Importantly, P7/8 are part of the network and overly areas that have visual functions.

By overlaying the connectivity structures of the theta and the alpha networks, the unique characteristics of the Pz node become visible—it acts as a sink. Additionally, the Pz is also distinctive because of its activity pattern, as the alpha (before and after the ‘alpha drop’) and theta oscillations have comparatively high amplitudes of about 10 µV. In contrast, at CPz only weak alpha activity and theta oscillations with similar amplitude values can be observed.

The tensor decomposition shows that the peak frequency of the frequency mode of factor #3 is located at a slightly higher frequency (12 Hz) than the corresponding peak of factor #5 (11 Hz); however due to the low frequency resolution of PDC one should be very careful when interpreting this finding. Factor #5 describes the alpha connectivity network during BSS; this is clearly shown by the time mode. This may indicate that the alpha network during BUS is not an expanded variant of the network which can be observed during BSS, i.e. different visual information processing and connectivity properties could be assumed for both situations.

### Methodological Considerations

The occurrence of strong movement-related artifacts limits the interpretation of our results. The state-of-the-art techniques for the rejection of such artifacts are ICA-based (Gwin et al. 2010). We have previously shown that not only the artifact itself, but also artifact rejection can influence the results of connectivity analysis. Thus, if a selection of artifact-free analysis intervals and/or frequency ranges is possible, then such a selection-based strategy should be preferred. By using time-variant PDC approaches artifact contaminated time intervals and/or frequency ranges can be excluded from interpretation, but some residual uncertainty from artifactual influences remains. A further uncertainty results from the frequency resolution of all PDC approaches (time-invariant and time-variant), which is lower than the frequency resolution of a corresponding spectral analysis based on the same multivariate model (time-resolutions are comparable).

The discretization of PDC values is a necessary step to construct visually assessable weighted directed networks. We used different discretization schemes, where the fundamental network structures and their time evolution remain.

One observation during BUS must be mentioned within the framework of a critical discussion of methodology. That is, the strengths of the interactions from Fp1 and Fp2 to the theta network electrodes (‘showerhead-like’ sources of interactions) decrease to the degree that the interaction strength of the theta network increases. Therefore, the strength gain could be partially caused via the tvMVAR model. The minor extent of such a hypothesized methodological contribution can be demonstrated by the finding that the corresponding network sequence on the basis of the reduced tvMVAR (SI 2.3B) shows a similar structure and also a gain of interactions over time.

Additionally, the influence of filtering for data downsampling (see “Methods ” section) must be considered. The possible effects can be discussed by results of two studies which used different filter settings to investigate their influence on time-invariant (Florin et al. 2010) and time-variant PDC (Leistritz et al. 2013). For the Matlab decimate procedure (downsampling from 1000 to 100 Hz, i.e. the upper frequency limit is 50 Hz) a low pass Chebyshev Type I IIR filter of order eight is used. Our own investigations concerning low pass filter influences on time-variant PDC (Leistritz et al. 2013; Pester 2016) have shown that ratios of the upper frequency limit (filter) and the frequency peak of the signal higher than two are less relevant, in particular when using IIR filter. The ratio values for downsampling are higher (theta: 8.33; alpha: 5), i.e. influences are rather unlikely.

Finally, a notable point is that all analyses have been performed in sensor space. We were aware that an ‘interpretation of estimated connectivity from sensor level recordings is not straightforward, as these recordings are severely corrupted by effects of field spread’ (Schoffelen and Gross 2009), i.e. that results obtained from sensor space analyses should be discussed more cautiously than results from source space. Source space connectivity analyses by using our time-variant PDC computation were successfully performed (Astolfi et al. 2008, 2010; Fallani et al. 2008; Leistritz et al. 2016), notably with a high number of electrodes and an individual, realistic head model. Particularly these two prerequisites are still considered as basic requirement for sufficiently accurate connectivity analysis in source space. However, there are some known, unresolved issues which were emphasized once again by current studies. Problems caused by source mixing due to volume conduction arise not only in sensor space (Leistritz et al. 2013), but also in source space (Haufe and Ewald 2016). There are altogether three methodological issues which must be taken into consideration for source connectivity analysis (Hassan et al. 2014; Leistritz et al. 2016): (1) the solution of the inverse problem is ill-posed, (2) an appropriate source and head model as well as connectivity measure must be chosen, and (3) volume conduction effects can never be completely abolished. Furthermore, in their recent work, Blinowska and colleagues argue that ‘pre-processing such as Hjorth transform or projection into source space involves mathematical operations that mix the information from the signals of the set, so the phase information is lost. Therefore, this kind of pre-processing should be avoided’ (Kaminski et al. 2016). This is important because causal information is coded in the delays between given signals (Kaminski et al. 2016). This discussion concerning reliable analysis and interpretation gave the impetus for the development of evaluation and benchmarking strategies (e.g. Haufe and Ewald 2016), which will be helpful to improve analysis strategies and/or the interpretation of results.

## Conclusions

It can be concluded that in humans at least two topographically delimitable functional cortical networks could contribute to an optimization of balance control. The theta network is suggested to facilitate somatosensory information processing and integration as well as the planning and execution of required motor responses. The alpha network encompasses the assumed ventral and dorsal pathways, and is suggested to promote visual information processing necessary for optimal body stability. Our results reveal that PDC is capable of minimizing the influence of movement-related artifacts which makes it a particularly valuable analysis tool for mobile EEG research. The application of tensor decomposition offers important additional information on an individualized level for each subject.

## Electronic supplementary material

Below is the link to the electronic supplementary material.

Supplementary material 1 (DOCX 425 KB)
