# Supplementary material for: Cortical Correlates of Human Balance Control
Source: Brain Topogr. 2017 May 2;30(4):434–46. doi: 10.1007/s10548-017-0567-x (PMC5495870; doi:10.1007/s10548-017-0567-x)
Supplement: Supplementary file 1 — Supplementary material 1 (DOCX 425 KB) [file 10548_2017_567_MOESM1_ESM.docx]

**Supporting Information**

# SI 1


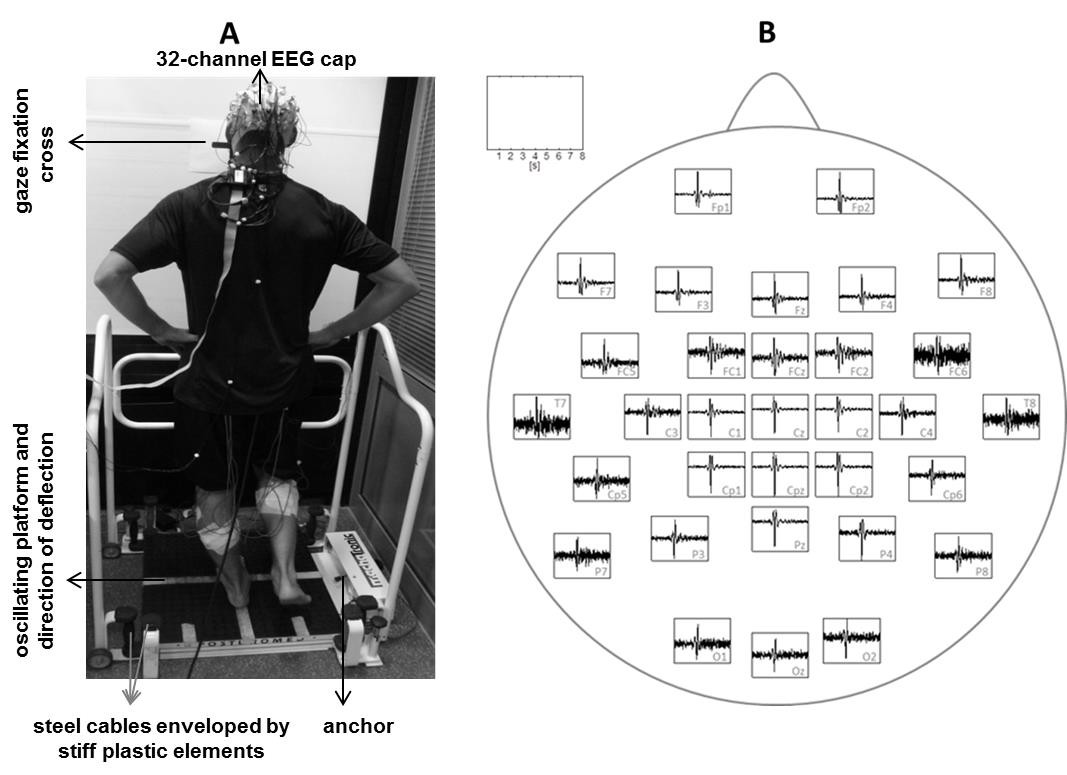


**Fig. SI 1. Experimental setup (A) and grand mean time series for each electrode (B).**

# SI 2 – Preliminary investigations

# Detection of artifacts

The experimental conditions give rise to the assumption that artifactual contaminations of the EEG signals occur, which can be attributed to e.g. mechanical electrode artifacts associated with head movement and cable sway (electromagnetic induction) caused by body movement. Such artifacts can have an amplitude that is in an order of magnitude larger than the brain related EEG signals (Gwin et al. 2010). Additionally, the vestibule-ocular reflex causes compensatory eye movements which occur conversely to head movements. Therefore, in our experiment (part of the task is to fixate a crosspoint during balancing) additional movement-related EOG interferences can be expected, particularly in the EEG of the fronto-polar electrodes (Fp1 and Fp2). Thereby those EEG components must be identified which can be attributed to the artifacts due to the experimental conditions (‘sway- related’ artifacts). Consequently, prior to connectivity analysis, we performed a thorough time-frequency analysis of the EEG data in order to identify the occurrence and possible influences of artifacts.

First we considered the artifactual influence of the platform oscillations which were calculated by a summation of the medio-lateral and anterior-posterior oscillation (Hülsdünker et al. 2015).

Therefore, we calculated the mean oscillation frequency which is 𝑓𝑜 = 2.6 Hz and has properties of a

damped oscillation. For this reason, the delta range must be excluded from the interpretation of the

EEG analysis results.

Secondly, we identified those electrodes which were severely contaminated by ‘sway-related’ and other artifacts. For this purpose, a time-variant spectral analysis using the matched Gabor transform

(MGT) was conducted (Wacker and Witte 2011). The interference around 𝑓𝑜 = 2.6 Hz is strongest at electrode sites Fp1 and Fp2, which may contaminate the lower theta band. Although we used standard eye movement artifact rejection, additional artifacts caused by compensatory eye movements (oculovestibular reflex) cannot be prevented. Consequently, the electrode sites Fp1 and Fp2 should be excluded from the interpretation (not from the multivariate AR model, see below). Additionally, electrode sites T7, T8, P7, and P8 should be interpreted very carefully. For Fp1 and Fp2 the contamination is caused from the ‘sway-related’ interference in the delta band and extends to the alpha band.

Thirdly, the EEG frequency range used in the analysis must be examined for possibly occurring higher harmonics of the ‘sway-related’ interference, which can be expected for (non-sinusoidal) periodic signals at frequencies 𝑘 ∙ 𝑓𝑜 = 𝑘 ⋅ 2.6 Hz (with 𝑘 = 1,2, …), i.e. the theta and alpha range may be contaminated. The ‘sway-related’ interference is observed to varying degrees in all EEG signals and it is phase-locked to the onset of balancing on the BUS condition, i.e. possibly occurring higher harmonics must also be phase-locked. This means that phase-locked oscillations must be detected in order to avoid misinterpretations. This can be performed using the time-variant phase-locking index (PLI). The PLI is amplitude-independent and is able to detect phase-locked oscillation, i.e. also their harmonics (Wacker et al. 2011). Our corresponding analysis concept encompasses the computation of the TFMs by using the time-variant multivariate autoregressive (tvMVAR) model, which is used in the PDC-TFM computation, as well as the MGT approach.

Additionally, the MGT-based PLIs were computed. In Fig. 2C of the article main body the PLI TFMs for the electrodes CPz and O2 are depicted; CPz is the central node of the ‘theta network’ and O2 is a node of the ‘alpha network’ in our connectivity analysis. In row A the amplitude TFMs derived from the tvMVAR parameters and in row B TFMs computed by the MGT are shown. It can be demonstrated that the time-frequency resolution (TFR) achieved by the tvMVAR approach (Fig. 2A) is comparable with the TFR from the MGT (Fig. 2B), i.e. that our tvMVAR model is adapted to the data in the best way possible. The TFR of the tvMVAR model is constant (frequency - independent) and that from the MGT is optimal for each frequency component (gold standard with regard to TFR). The center value of the ‘sway-related’ component is at 2.6 Hz, i.e. the 2nd harmonics would occur at 5.2 Hz, the 3rd at 7.8 Hz , and the 4th at 10.4 Hz (red horizontal lines). However, the PLI TFMs in Fig. 2C clearly show that there are no indications for the occurrence of (phase-locked) higher harmonics (which would be reflected by traces in the PLI maps). The interference at 2.6 Hz is significantly phase-locked (significance level 0.05) within the interval 3 – 6 s, no other phase-locked components (e.g. higher harmonics) can be detected. At the onset of the BUS task a short and strong displacement artifact can be observed in all channels, i.e. the time interval 3 – 3.5 s should not be considered in the connectivity analysis.

The TFM analysis shows that theta oscillations, i.e. circumscribed traces in the theta band, can only be detected at electrode sites CPz, Pz, CP1, CP2, and FC1, where theta amplitude is highest at CPz and Pz (see example for CPz in Fig. 2A/B). The trace of the theta oscillation is located at about 6 Hz. Consequently, we used 𝜃ROIs in the frequency range 5 – 7 Hz, i.e. 6 Hz is the mid-frequency of the band, with enough distance to the beta (artifactual) and alpha band activity. Continuous alpha activity can be shown during both balancing conditions at the occipital and parietal electrodes, which is interrupted by an alpha amplitude suppression (‘alpha drop’) occurring after the beginning of the BUS task (duration about 1 s). The alpha trace is located at about 10 Hz, therefore we used the sub-band 9 – 11 Hz for the definition of our alpha ROIs. It can be shown that a strong alpha amplitude suppression occurs (O1/2/z; for O2), where the suppression maximum is reached 1 s after the onset (at 4 s). The alpha-amplitude is returned to its starting level around 2 s after the onset. This alpha drop can be observed in a weaker form at the parietal electrodes. After the ‘alpha drop’ the alpha amplitudes at the occipital electrodes are slightly increased.

# Influence of remaining artifacts on the results of connectivity analysis

The influence of strongly artifact-contaminated electrodes on the results of connectivity analysis in the theta and alpha frequency range was tested. For this the PDC-TFMs based on the full (EEG from all electrodes) and the reduced (without the EEG from the contaminated electrodes) tvMVAR models were computed and the resulting networks were compared. It can be shown that in particular the fronto-polar electrodes act only as a source (like a ‘shower head’) with interactions up to anterior areas. This can be demonstrated by the theta network structures derived from theta ROI 1 and 4 (Fig. SI 2.1A). If the two fronto-polar electrodes are rejected from the tvMVAR model (Fig. SI 2.1B) then the sub-network structure changes only marginally (Fig. SI 2.1 colored area). The interactions from F7 to C2 and CP2 (Fig. SI 2.1B, TI1) are possibly carried over from the corresponding Fp1 interactions. Otherwise, only the level of interaction strengths (weights of edges) is slightly increased, i.e. the fixed discretization limits cause the majority of marginal changes. Important for the analysis of theta networks structures is that the interactions from all artifactual electrodes (Fp1/2, T7/8, P7/8) do not reach the centro-parietal, parietal, and occipital electrodes, i.e. the structure and structural changes of these sub-networks can be reliably interpreted. All results indicate that the full tvMVAR model can be used, i.e. the electrodes Fp1 and Fp2 must only be rejected from the interpretation (network representations) not from the modeling. A comparison of the results of connectivity analysis using the full and reduced tvMVAR was also performed using the alpha sub-band 9 - 11 Hz. For this band the influence of both electrodes on the network structure is even smaller. For the BSS period (intervals 0.5 – 1.5 s and 1.5 – 2.5 s) mean PDC values > 0.1 only between the occipital electrodes can be observed.


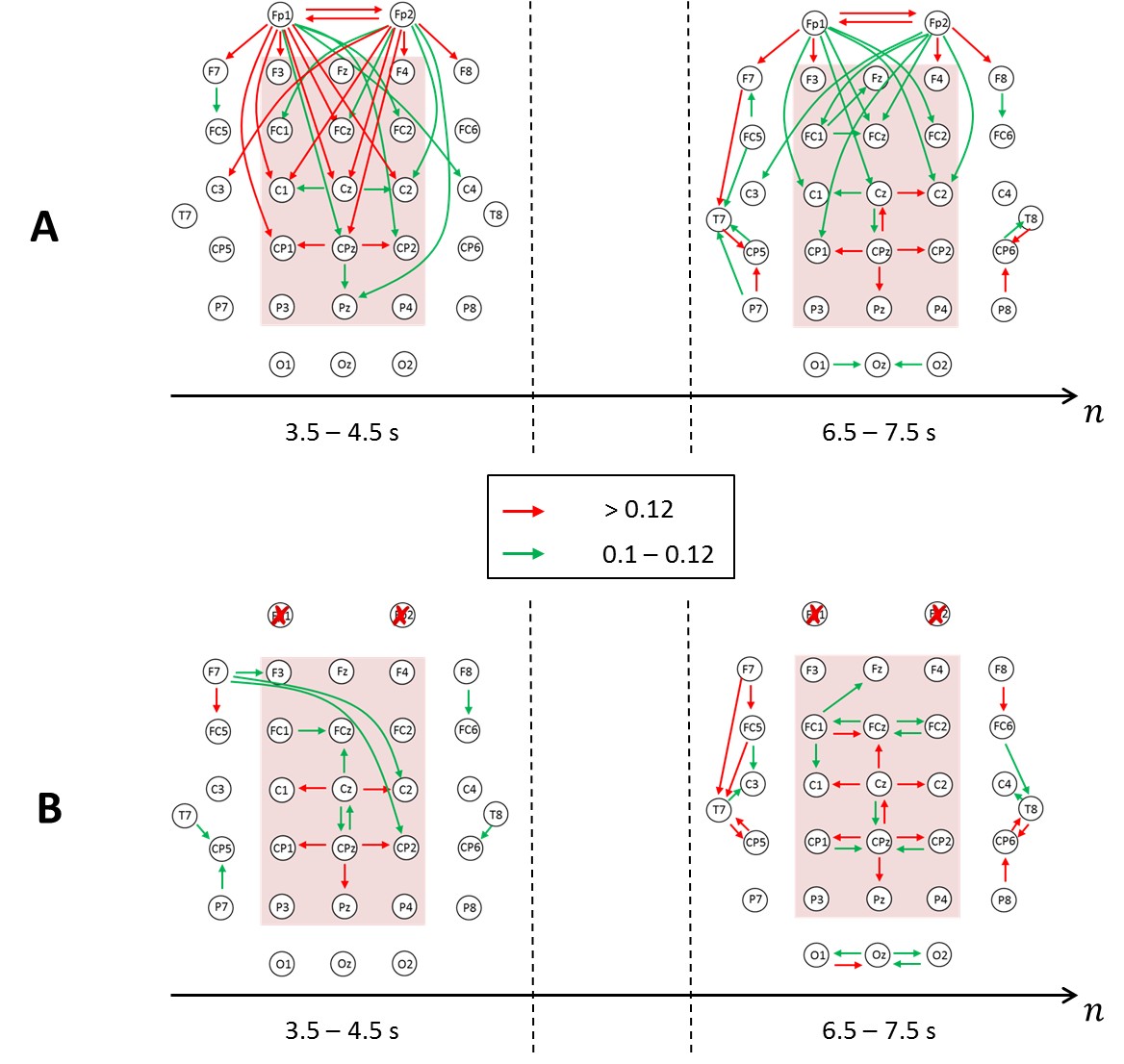


**Fig. SI 2.1. Network structures (grand mean) of theta ROI 1 and 4 by using a full (A) and a reduced (B) tvMVAR. Only the strongest interactions are depicted (two discretization steps for the mean PDC). To gain a better overview we have depicted only the two strongest interactions (discretization steps). Colored area indicates relevant sub-network.**

# Statistical analysis of frequency band activity

Fifth, after the artifact-related investigations a statistical analysis of the frequency band activity between BSS and BUS situation was carried out. In particular, at CPz the mean amplitude within the theta sub-band is increased 2 s after the onset (from about 7.5 to 10 µV), after the slowdown of the artifact influence. The time-courses of the mean spectral amplitude in two specific frequency sub-bands were extracted (theta sub-band 5 – 7 Hz and alpha sub-band 9 – 11 Hz) for each subject and each electrode. In order to estimate confidence tubes of the mean time courses of extracted parameters without any particular distribution assumption, a Bootstrap approach was used. For each electrode (*D*=32), one thousand Bootstrap samples of size 37 (37 subjects; each sample element contains 800 values/8 s) were drawn by a case resampling with replacement. 1000 Bootstrap replications of each extracted parameter were then computed. Based on these replications, the lower limit (the 2.5% quantile) defined the lower bound, and the upper limit (the 97.5% quantile) defined the upper bound of the confidence tube. The computation of these confidence tubes (95% confidence intervals for each sampling point and each electrode) allows a statistical quantification of the differences for all time-points, i.e. the mean amplitude values of two sampling points are significantly different when the corresponding 95%-confidence intervals are non-overlapping. Analogous to Fig. 2, examples of results are provided for CPz and O2 in Fig. SI 2.2. The red horizontal line denotes the maximal value of the confidence tube (maximum of the upper limit of the confidence tube) during BSS; therefore, any mean amplitude in a specific frequency band during BUS is significantly different from BSS if the corresponding value of the lower limit of the confidence tube is above this line. According to the theta confidence tube in Fig. SI 2.2A, the mean theta amplitude is significantly increased during BUS in comparison to the BSS condition for electrodes CPz and Pz (5 – 8 s) as well as Cz, C1, CP1, CP2 (5 – 6 s). According to the confidence tube analysis, a significant difference of the alpha amplitude between BSS and BUS cannot be observed (example in Fig. SI 2.2B).


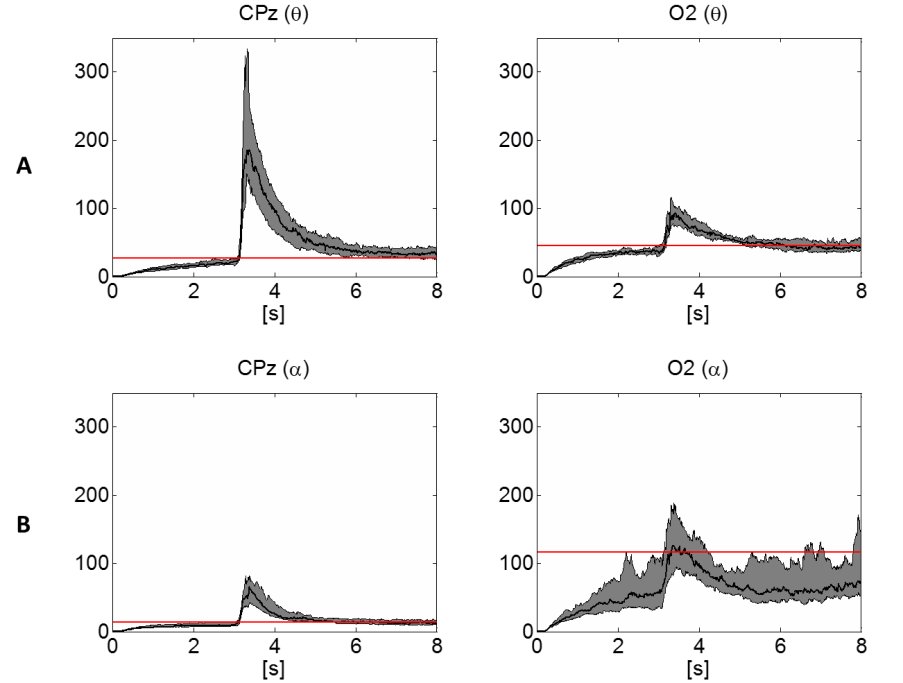


**Fig. SI 2.2. Mean time-courses and corresponding 95% confidence tubes of the mean spectral amplitude in a specific frequency sub-band of electrodes CPz and O2. (A) Results of theta sub-band (5 – 7 Hz); the red line denotes the upper limit of the confidence tube during BSS. Therefore, every value that is below this line can be considered as significantly different from BUS. (B) Results for the alpha sub-band (9 – 11 Hz). The change from BSS to BUS occurred at time 3 s.**

# Summary of preliminary investigations

Based on the above descriptions, the findings can be summarized as follows: (1) for the theta (5 – 7 Hz) and the alpha (9 – 11 Hz) band a contamination by higher harmonics of the ‘sway-related’ interference is unverifiable, so that a connectivity analysis is meaningful. (2) The time-interval ranging from 3 to 3.5 s is strongly influenced by the BUS onset (MGT analysis). This is also true for the theta and alpha bands. According to the time-resolution of the tvMVAR-based analysis, the artifact influence is detectable until 2 s after the BUS onset, i.e. only the networks for the ROIs2/3/4 (𝜃 and 𝛼) are reliable. (3) The PDC results which are related to the electrode sites Fp1 and Fp2 should be excluded from an interpretation and those related to the sites T7, T8, P7, and P8 must be interpreted carefully. (4) The influence of artifactual EEG activity on the central, centro-parietal, parietal and occipital network structures (interactions) is only marginal. Therefore the full tvMVAR model is used for further investigations. (5) The mean amplitude differences between BSS and BUS are particularly significant for the theta sub-band 5 – 7 Hz at the central, centro-parietal, and parietal midline electrodes and for the alpha sub-band 9 – 11 Hz at the occipital electrodes during the ‘alpha drop’.
